# Impact of an SMS intervention to support type 2 diabetes self-management: DiabeText clinical trial

**DOI:** 10.3399/BJGP.2024.0206

**Published:** 2025-06-27

**Authors:** Rocío Zamanillo-Campos, María Antonia Fiol-deRoque, María Jesús Serrano-Ripoll, Joan Llobera-Canaves, Joana María Taltavull-Aparicio, Alfonso Leiva-Rus, Joana Ripoll-Amengual, Escarlata Angullo-Martínez, Isabel María Socias-Buades, Lluís Masmiquel-Comas, Jadwiga Konieczna, María Zaforteza-Dezcallar, María Asunción Boronat-Moreiro, Sofía Mira-Martínez, Elena Gervilla-García, Ignacio Ricci-Cabello

**Affiliations:** Research Group on Primary Care and Promotion of the Balearic Islands Community (Grapp-CAIB), Health Research Institute of the Balearic Islands (IdISBa), Palma, and Primary Care Research Unit of Mallorca, Balearic Islands Health Service, Palma; Research Group on Primary Care and Promotion of the Balearic Islands Community (Grapp-CAIB), Health Research Institute of the Balearic Islands (IdISBa), Palma, and RICAPPS — Red de Investigación Cooperativa de Atención Primaria y Promoción de la Salud, Carlos III Health Institute (ISCIII), Madrid; Research Group on Primary Care and Promotion of the Balearic Islands Community (Grapp-CAIB), Health Research Institute of the Balearic Islands (IdISBa), Palma, and RICAPPS — Red de Investigación Cooperativa de Atención Primaria y Promoción de la Salud, Carlos III Health Institute (ISCIII), Madrid; Research Group on Primary Care and Promotion of the Balearic Islands Community (Grapp-CAIB), Health Research Institute of the Balearic Islands (IdISBa), Palma, and RICAPPS — Red de Investigación Cooperativa de Atención Primaria y Promoción de la Salud, Carlos III Health Institute (ISCIII), Madrid; Research Group on Primary Care and Promotion of the Balearic Islands Community (Grapp-CAIB), Health Research Institute of the Balearic Islands (IdISBa), Palma, and Primary Care Research Unit of Mallorca, Balearic Islands Health Service, Palma; Research Group on Primary Care and Promotion of the Balearic Islands Community (Grapp-CAIB), Health Research Institute of the Balearic Islands (IdISBa), Palma, and RICAPPS — Red de Investigación Cooperativa de Atención Primaria y Promoción de la Salud, Carlos III Health Institute (ISCIII), Madrid; Research Group on Primary Care and Promotion of the Balearic Islands Community (Grapp-CAIB), Health Research Institute of the Balearic Islands (IdISBa), Palma, and Primary Care Research Unit of Mallorca, Balearic Islands Health Service, Palma; Research Group on Community Nutrition and Oxidative Stress (NUCOX), Health Research Institute of the Balearic Islands (IdISBa), Palma, and Primary Care Health Services of the Balearic Islands (IbSalut), Palma; Primary Care Health Services of the Balearic Islands (IbSalut), Palma; Vascular and Metabolic Pathologies Group, Health Research Institute of the Balearic Islands (IdISBa), Palma, and Endocrinology and Nutrition Department, Son Llàtzer University Hospital of the Balearic Islands (IbSalut), Palma; Research Group on Nutritional Epidemiology and Cardiovascular Physiopathology (NUTRECOR), Health Research Institute of the Balearic Islands (IdISBa), Palma, and CIBER de Fisiopatología de la Obesidad y Nutrición (CIBERobn), Instituto de Salud Carlos III, Madrid; Pharmacy Service, Balearic Islands Health Service, Palma; Pharmacy Service, Balearic Islands Health Service, Palma; Research Group on Primary Care and Promotion of the Balearic Islands Community (Grapp-CAIB), Health Research Institute of the Balearic Islands (IdISBa), Palma, Spain, and Primary Care Research Unit of Mallorca, Balearic Islands Health Service, Palma; Statistical and Psychometric Procedures Applied in Health Sciences (PSICOMEST), Health Research Institute of the Balearic Islands (IdISBa), Palma, and Data Analysis Research Group (GRAD), Psychology Department, University of the Balearic Islands (UIB), Palma; Research Group on Primary Care and Promotion of the Balearic Islands Community (Grapp-CAIB), Health Research Institute of the Balearic Islands (IdISBa), Palma, and CIBER de Epidemiología y Salud Pública (CIBEResp), Instituto de Salud Carlos III, Madrid

**Keywords:** glycaemic control, medication adherence, primary care, quality of life, telemedicine, type 2 diabetes mellitus

## Abstract

**Background:**

Complications arising from uncontrolled type 2 diabetes mellitus (T2DM) pose a significant burden on individuals’ wellbeing and healthcare resources. Digital interventions may play a key role in mitigating such complications by supporting patients to adequately self-manage their condition.

**Aim:**

To assess the impact of DiabeText, a new theory-based, patient-centred, mobile health intervention integrated with electronic health records to send tailored short text messages to support T2DM self-management.

**Design and setting:**

A pragmatic, phase-three, 12-month, two-arm randomised clinical trial involving primary care patients with T2DM in the Balearic Islands, Spain, including in urban and rural areas.

**Method:**

In total, 742 participants with suboptimal glycaemic control (glycated haemoglobin [HbA1c] level >7.5) were randomly allocated to a control (usual care) or intervention (DiabeText) group. In addition to usual care, the DiabeText group received 167 messages focused on healthy lifestyle and medication adherence. The primary outcome was HbA1c level. Secondary outcomes were: medication possession ratio; health-related quality of life (measured using the EQ-5D-5L questionnaire); diabetes self-efficacy (measured using the Diabetes Self-Efficacy Scale [DSES]); and self-reported adherence to medication, Mediterranean diet (measured using the 14-Item Mediterranean Diet Adherence Screener [MEDAS-14]), and physical activity (measured using the International Physical Activity Questionnaire [IPAQ]).

**Results:**

Over the 12-month period, no statistically significant differences in HbA1c were observed between the intervention and the control groups (β = −0.025 [95% confidence interval {CI} = −0.198 to 0.147; *P* = 0.772]). In comparison with the control group, the DiabeText group showed significant (*P*<0.05) improvements in self-reported medication adherence (odds ratio = 1.4; 95% CI = 1.0 to 1.9), DSES (Cohen’s *d* = 0.35), and EQ-5D-5L (Cohen’s *d* = 0.18) scores, but not for the rest of the secondary outcomes.

**Conclusion:**

DiabeText successfully improved quality of life, diabetes self-management, and self-reported medication adherence in primary care patients with T2DM. Further research is needed to enhance its effects on physiological outcomes.

## Introduction

Type 2 diabetes mellitus (T2DM) is a condition that contributes to the top causes of premature death.^1^ People with T2DM are at high risk of developing serious complications (for example, blindness, lower-limb amputations, kidney disease, and cardiovascular disease), which reduce their quality of life and life expectancy.^2^ The cornerstone of T2DM management is promoting a healthy lifestyle^3^ and taking glucose-lowering drugs,^4^ but adherence to treatment plans is the true bottleneck for achieving good glycaemic control in patients with T2DM.^5^–^7^

Available evidence from recent meta-analyses suggests that educational and behavioural diabetes self-management interventions can effectively reduce HbA1c by ~0.6%.^8^–^10^ Over the last decade, advances in technology and connectivity have led to a boom of mobile health (mHealth) interventions, as they constitute a low-cost and highly scalable approach to support behaviour changes in terms of lifestyle^11^ and medication taking.^12^ However, as highlighted by the European Association for the Study of Diabetes and the American Diabetes Association,^13^ although available studies of mHealth interventions show there is promise in terms of promoting healthy behaviour and controlling HbA1c, they are very limited in quantity and quality. According to the most recent systematic reviews and meta-analyses, there is a pressing need for large, adequately powered randomised controlled trials (RCTs) to examine longer-term efficacy,^14^ before the adoption and widespread dissemination of such interventions is promoted.^15^,^16^

**How this fits in**

Previously, it was known that digital interventions could support diabetes self-management, but their impact on clinical outcomes, such as glycaemic control, was inconsistent. This study adds evidence that the DiabeText mobile health intervention, when integrated with electronic health records, greatly improved self-reported medication adherence, diabetes self-efficacy, and health-related quality of life in patients with type 2 diabetes. However, it did not lead to statistically significant changes in glycaemic control, medication possession ratio, or lifestyle behaviours over a 12-month period. These findings highlight the importance of digital interventions in enhancing patient-reported outcomes — which are crucial for clinicians aiming to improve overall patient wellbeing — despite the challenges in affecting physiological measures.

To contribute to making progress in this area, the authors developed DiabeText, a new theory-based, patient-centred, mHealth intervention integrated with electronic health records (EHRs) to send tailored short text messages (SMSs) to support patients with T2DM. DiabeText was developed following the Medical Research Council guidelines in a process that involved three systematic reviews^17^–^19^ and two formative qualitative studies — one with patients^20^ and one with primary care providers.^21^ In a phase-two feasibility trial,^22^ the authors observed that integrating DiabeText with EHRs and patient-generated data was feasible for delivering tailored, meaningful SMSs to patients with T2DM; they also found that DiabeText may be effective in supporting lifestyle changes and medication adherence.

This phase-three trial aimed to assess the long-term effectiveness of DiabeText. The authors hypothesised that, compared with standard care over a 12-month period, the DiabeText intervention would reduce HbA1c by ≥4 mmol/mol (0.4%) in patients with T2DM.

## Method

### Design

This 12-month, phase-three parallel (1:1 allocation ratio) RCT with patients with T2DM was conducted in the Balearic Islands, Spain. The reporting of this manuscript adheres to the CONSORT 2010 guidelines.^23^ The study is registered at ClinicalTrials.gov (identifier: NCT05006872) and the protocol has been published previously.^24^

### Study participants

Patients were included if they:

- had T2DM registered in the public health service of the Balearic Islands;
- were aged >18 years (there was no upper age limit);
- had had an HbA1c level of >7.5% recorded during the previous 6 months;
- had at least one prescription of a non-insulin antidiabetic drug; and
- were able to receive, read, and understand an SMS in Spanish through a personal mobile phone or they had a caregiver who could do it for them.

People with severe mental health conditions or those who were participating in other research studies were excluded.

### The DiabeText intervention

For 12 months, participants allocated to the intervention (DiabeText) group were sent 167 SMSs, distributed as follows:

- five SMSs per week for 4 months;
- three SMSs per week for the next 4 months; and
- two SMSs per week for the last 4 months.

All the participants received messages about four key areas: medication, diet, physical activity, and diabetes self-management. The proportion of messages about diet and physical activity was modulated based on patient-reported lifestyle behaviour (details are available in Supplementary Table S1). Participants received additional messages with their latest blood test results, changes in body weight, and reminders for medical appointments and pharmacy medication pick-ups when this new information was recorded in their EHRs.

### Sample size

The necessary sample size was estimated at 740 participants (370 per group) to detect changes in HbA1c of ≥4 mmol/mol (0.4%) between groups based on a standard deviation (SD) of 15 mmol/mol (1.5%). This estimate includes a 20% loss to follow-up at 90% power and a *P*-value of 0.05.

### Recruitment and baseline data collection

With support from an information specialist, the authors extracted a list of patients who potentially met the eligibility criteria, based on data recorded in Sistema de Información de Atención Primaria (Primary Care Information System) (e-SIAP), the primary care EHR system for the Balearic Islands. In total, 2218 potential participants were sent an SMS inviting them to participate in the study. The SMS included a link to the study information document. Subsequently, trained research assistants telephoned them to seek confirmation of eligibility criteria and to record informed consent when appropriate. Baseline data were then collected through semi-structured telephone interviews.

### Randomisation and blinding

Randomisation was performed using an adaptation of the OxMaR software; details of the adaptation have been previously published.^25^,^26^ A non-deterministic minimisation algorithm was applied to ensure the groups were balanced on important baseline prognostic factors (age and sex). Patients were informed about their allocation to the control (receiving usual care) or the intervention (usual care plus DiabeText) group only after all baseline data had been collected. Data collectors and analysts were blinded.

### Outcomes

The primary outcome was HbA1c level (%) at 12-month follow-up (post-intervention). Secondary outcomes included: medication possession ratio (MPR);^27^ self-reported adherence to diabetes medication;^28^ health-related quality of life (HRQL), measured using the EQ-5D-5L questionnaire;^29^,^30^ diabetes self-efficacy, measured using the Diabetes Self-Efficacy Scale (DSES);^31^ adherence to Mediterranean diet, measured using the 14-Item Mediterranean Diet Adherence Screener (MEDAS-14);^32^ and physical activity, measured using the International Physical Activity Questionnaire (IPAQ).^33^ *Ad-hoc* questionnaires were used to measure users’ satisfaction, perceived utility, perceived ease of access, and potential harms related to the intervention. A detailed description of outcomes measures and methods for data collection is given in Supplementary Box S1.

Mean and SDs or median and interquartile range (IQR) (as appropriate) were used for continuous variables, while frequencies and percentages were used for categorical variables in the descriptive analysis of the participants’ sociodemographic, lifestyle, and health-related characteristics. The effect of the intervention was evaluated using general linear models or logistic regression as appropriate, adjusted for corresponding baseline values. The adjusted mean difference between groups is presented along with its associated 95% confidence interval (CI) and *P*-value. Cohen’s *d*^34^ was calculated to estimate effect sizes. The main analyses were carried out on the basis of intention to treat, using multiple imputation chained equations models with 10 iterations^35^ and bootstrap inference.^36^

As a sensitivity analysis, all outcomes were reanalysed on a complete-case basis (that is, without imputation). Additionally, a sensitivity analysis was conducted that excluded participants with an HbA1c level of <8% at baseline. Subgroup analyses (statistical tests for interaction) were conducted based on sex (men versus women), age (<65 years versus ≥65 years old), medication taking at baseline (non-adherent versus adherent), and participant-reported use of internet on their mobile phones (yes versus no). All analyses were carried out in Stata 15 (StataCorp) and an α of 5% was used throughout.

### Deviation from the protocol

During the recruitment of participants, it was deemed necessary to reduce the HbA1c cut-off from 8.0% to 7.5% to achieve the target sample size for this trial.

## Results

### Recruitment, intervention reach, and baseline characteristics

**Figure 1:**
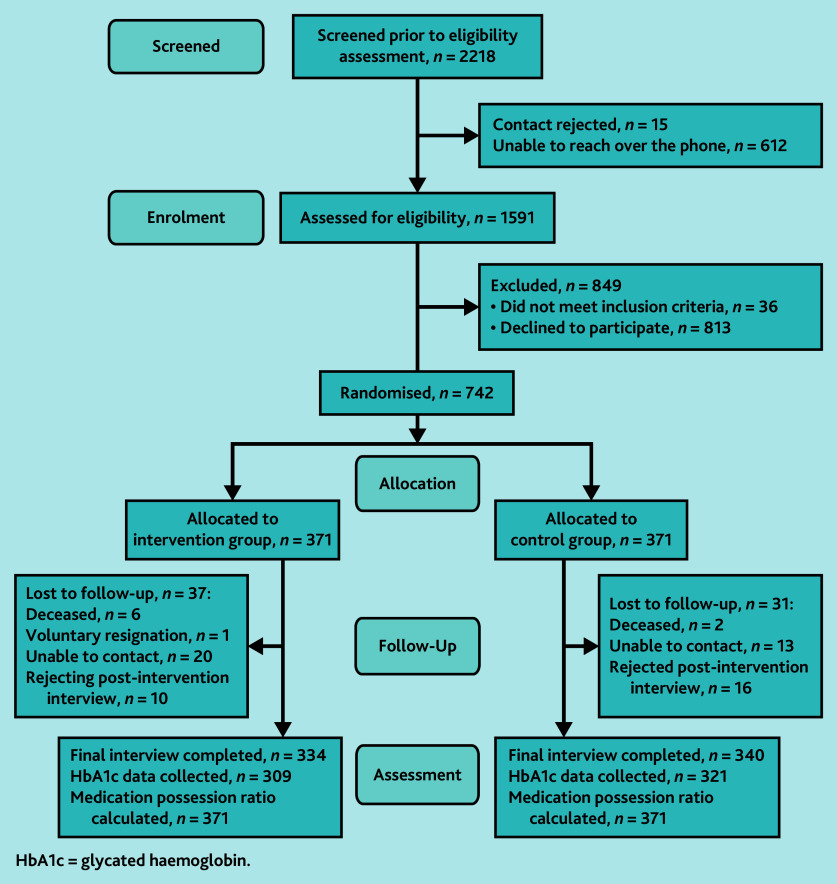
CONSORT flow diagram in the DiabeText randomised controlled trial. HbA1c = glycated haemoglobin.

Between 6 October and 18 November 2021, SMS invitations were sent to 2218 potentially eligible participants. Among the 1591 patients who were successfully contacted via telephone and confirmed as meeting the eligibility criteria, 742 agreed to participate (recruitment rate = 47%). At 12 months, a total of 68 patients had withdrawn or been lost to follow-up (withdrawal rate = 9.2%); 674 patients (340 in the control group and 334 in the intervention group) completed the post-intervention assessment (Figure 1). Regarding the intervention reach, most participants in the intervention group (95.2%) received >90% of the DiabeText messages intended to be delivered as part of the intervention (>165 SMS in 1 year).

The baseline sociodemographic and clinical characteristics of the 742 participants are shown in Table 1. In total, 42% were women (309/742), with a mean age of 65 (SD = 10) years and 10 (SD = 5) years since the diagnosis of T2DM. More than a half of participants (*n* = 428, 58%) had obesity (BMI >30), around two-thirds (*n* = 513, 69%) had hypertension, and just under one-quarter were current smokers (*n* = 178, 24%). The mean number of active antidiabetic prescriptions was 1.72 (SD = 0.71). The median HbA1c level was 8.1% (IQR = 7.7 to 8.8%) (data not shown). No relevant differences were observed in baseline sociodemographic and clinical characteristics between the intervention and control groups (Table 1); in addition, no relevant differences were seen between participants with complete or incomplete follow-up data (data not shown).

**Table 1:** Baseline sociodemographic and clinical characteristics of all participants (*n* = 742), and by control (*n* = 371) and intervention (*n* = 371) groups

	All participants	Control group	Intervention group
**Sociodemographic characteristics**			
Female, *n* (%)	309 (42)	154 (42)	155 (42)
Age in years, mean (SD)	65 (10)	65 (10)	65 (10)
Current smoker, *n* (%)	178 (24)	84 (23)	94 (25)
Reduced mobility, *n* (%)	24 (3)	8 (2)	16 (4)
Use of internet on mobile phone, *n* (%)	537 (72)	269 (73)	268 (72)

**Clinical characteristics**			
Diabetes duration in years, mean (SD)	10 (5)	10 (5)	10 (5)
Caregiver, *n* (%)	51 (7)	29 (8)	22 (6)
Number of antidiabetic active substances, mean (SD)	2 (1)	2 (1)	2 (1)
Number of antidiabetic prescriptions, mean (SD)	2 (1)	2 (1)	2 (1)
Number of overall prescriptions, mean (SD)	7 (4)	7 (4)	7 (4)
Hypertension, *n* (%)	513 (69)	250 (67)	263 (71)
Obesity: BMI >30, *n* (%)	428 (58)	219 (59)	209 (56)
Depression, *n* (%)	164 (22)	90 (24)	74 (20)
Diabetic foot, *n* (%)	64 (9)	33 (9)	31 (8)
Chronic kidney disease, *n* (%)	87 (12)	51 (14)	36 (10)
Diabetic retinopathy, *n* (%)	20 (3)	9 (3)	11 (3)
Diabetic neuropathy, *n* (%)	20 (3)	7 (2)	13 (4)

BMI = body mass index. SD = standard deviation.

### Glycaemic control

Follow-up HbA1c data were successfully collected from 630 (85%) of the 742 participants in the study. At baseline, no statistically significant differences were observed between the intervention (8.1% [IQR = 7.7 to 8.7]) and control group (8.0% [IQR = 7.6 to 8.8]). Over the 12-month period, there was a similar reduction in HbA1c levels in both groups (7.4% and 7.5% in the control and intervention group, respectively), with no statistically significant differences between groups observed (β = −0.025 [95% CI = −0.198 to 0.147; *P* = 0.772]) (Table 2).

**Table 2:** Association between the DiabeText intervention and glycaemic control, adherence to medication treatment, diabetes self-efficacy, and HRQL^a^

	Baseline		12-month follow-up		Association coefficient estimates for participants in the intervention group compared with controls		Effect size
	Control group, *n* = 371	Intervention, *n* = 371	Control, *n* = 340	Intervention, *n* = 334	β 95% CI	*P*-value^b^	Cohen’s *d*, 95% CI
**Glycaemic control, median Hba1c % (IQR)**	8.0 (7.6 to 8.8)	8.1 (7.7 to 8.7)	7.4 (6.7 to 8.3)^c^	7.5 (6.7 to 8.2)^c^	−0.025 (−0.198 to 0.147)	0.772	n/a
**Adherence to medication treatment: MPR %, median (IQR)**	96.2 (83.1 to 99.3)	95.1 (81.8 to 99.3)	91.0 (73.6 to 97.8)^d^	90.4 (76.2 to 97.7)^d^	−0.587 (−2.348 to 1.173)	0.513	n/a
**Diabetes Self-Efficacy Scale, median (IQR)**	6.9 (5.9 to 7.9)	6.9 (5.8 to 8.0)	8.0 (6.6 to 8.9)	8.6 (7.4 to 9.3)	0.544 (0.319 to 0.770)	**<0.001** e	0.35 (0.20 to 0.50)
**HRQL: EQ-5D index, median (IQR)**	0.93 (0.89 to 1.00)	0.93 (0.88 to 1.00)	0.97 (0.89 to 1.00)	1 (0.92 to 1.00)	0.015 (0.002 to 0.027)	**0.019** e	0.18 (0.04 to 0.32)

a Analyses were performed using linear mixed-effects models adjusted for baseline values with MI BOOT estimations.

b *P*-values and 95% CI based on percentiles using non-parametric bootstrap.

c Post-intervention HbA1c level available for 321 patients in the control group and 309 in the intervention group.

d Post-intervention MPR available for 371 patients in each group respectively, so no imputations were necessary.

e *P*<0.05.

HbA1c = glycated haemoglobin. HRQL = health-related quality of life. IQR = interquartile range. MPR = medication possession ratio.

### MPR and self-reported medication adherence

Follow-up MPR data were successfully collected for all the study participants (*n* = 742). At baseline, no statistically significant differences were observed between the intervention and control groups. Over the 12-month period, there was a reduction in MPR values in both groups from median 96.2% and 95.1% at baseline to 91.0% and 90.4% at 12 months in the control and intervention groups respectively (Table 2). No statistically significant differences between groups were observed at 12-month follow-up (β = −0.587 [95% CI = −2.348 to 1.173; *P* = 0.513]) (Table 2). Similarly, no statistically significant differences were observed after dichotomising MPR to compare adherence (MPR ≥80%) versus non-adherence (MPR <80%) (odds ratio [OR] = 0.986 [95% CI = 0.702 to 1.385, *P* = 0.936]) (Table 3).

**Table 3:** Association between the DiabeText intervention and MPR, self-reported adherence to antidiabetic medication, adherence to Mediterranean diet, and adherence to physical activity^a^

	Baseline		12-month follow-up		Association odds for participants in the intervention group compared with controls	

	Control,^b^ *n* (%)	Intervention,^c^ *n* (%)	Control,^d^ *n* (%)	Intervention,^e^ *n* (%)	OR (95% CI)	*P*-value^f^
**MPR** g						
**Non-adherent: MPR <80%**	84 (22.6)	89 (24)	113 (30.5)	116 (31.3)	1	
**Adherent: MPR ≥80%**	287 (77.4)	282 (76)	258 (69.5)	255 (68.7)	0.986 (0.702 to 1.385)	0.936

**Self-reported adherence to antidiabetic medication**						
**Non-adherent**	177 (47.7)	166 (44.7)	143 (42.1)	114 (34.1)	1	
**Adherent**	194 (52.3)	205 (55.3)	197 (57.9)	220 (65.9)	1.397 (1.027 to 1.901)	0.033^h^,^i^

**Adherence to Mediterranean diet**						
**Non-adherent: MEDAS ≤5**	90 (24.3)	100 (27.0)	171 (50.3)	163 (48.8)	1	
**Adherent: adherent: MEDAS >5**	281 (75.7)	271 (73.0)	169 (49.7)	171 (51.2)	1.10 (0.827 to 1.481)	0.493

**Adherence to physical activity**						
**Non-adherent: low level**	126 (34.0)	131 (35.3)	146 (43)	163 (48.8)	1	
**Adherent: moderate or high level**	245 (66.0)	240 (64.7)	194 (57)	171 (51.2)	0.824 (0.601 to 1.133)	0.227

a Analyses were performed using logistic regression adjusted for baseline values with MI BOOT estimations.

b *n* = 371.

c *n* = 371.

d *n* = 340.

e *n* = 334.

f *P*-values and 95% CI based on percentiles using non-parametric bootstrap.

g Post-intervention MPR available for 371 patients in each group respectively, so no imputations were necessary.

h Statistical significance: *P*<0.05.

i Sex interaction (*P* = 0.022): men OR = 1.89 (95% CI = 1.26 to 2.83), *P* = 0.002; women OR = 0.90 (95% CI = 0.55 to 1.46), *P* = 0.672.

MPR = medication possession ratio. OR = odds ratio.

Regarding self-reported adherence to antidiabetic medication, there was a statistically significant improvement in the intervention group compared with the control group (OR = 1.40 [95% CI = 1.03 to 1.90; *P* = 0.033]) (Table 3).

### Lifestyle behaviour, diabetes self-efficacy, and HRQL

Follow-up data on the remaining outcomes were successfully collected for 674 participants. At baseline, no statistically significant differences were observed between the intervention and control groups in terms of the proportion of participants with low adherence to Mediterranean diet (27% and 24%) and with low levels of physical activity (35% versus 34%) respectively. Over the 12-month period, no statistically significant differences were observed in adherence to the Mediterranean diet (OR = 1.10 [95% CI = 0.83 to 1.48; *P* = 0.493]) or in physical activity (OR = 0.82 [95% CI = 0.60 to 1.13; *P* = 0.227]) (Table 3).

In relation to diabetes self-efficacy at baseline, DSES scores did not present statistically significant differences between the intervention (6.9 [IQR = 5.8 to 8.0]) and control groups (6.9 [IQR = 5.9 to 7.9]) (Table 2). At 12-month follow-up, DSES scores were statistically significantly (*P*<0.001) higher in the intervention group (8.63 [IQR = 7.38 to 9.25]) than in the control group (8.0 [IQR = 6.63 to 8.88]) (Table 2). A Cohen’s *d* of 0.35 (95% CI = 0.20 to 0.50) indicated that the effect size was small (Table 2).

In relation to HRQL at baseline, EQ-5D scores were not statistically significantly different between the intervention (0.93 [IQR = 0.88 to 1.00]) and control groups (0.93 [IQR = 0.89 to 1.00]). However, at 12-month follow-up, EQ-5D scores were statistically significantly (*P*<0.05) higher in the intervention group (1.00 [IQR = 0.92 to 1.00]) than in the control group (0.97 [IQR = 0.89 to 1.00]). A Cohen’s *d* of 0.18 (95% CI = 0.04 to 0.32) indicated a small effect size in HRQL (Table 2).

### Subgroup analysis

A significant (*P* = 0.022) interaction with sex was found for self-reported medication adherence, showing that DiabeText was associated with higher self-reported adherence in men (OR = 1.89 [95% CI = 1.26 to 2.83; *P* = 0.002]) but not in women (OR = 0.90 [95% CI = 0.55 to 1.46; *P* = 0.672]) (Table 3). No additional statistically significant subgroup interactions were observed in any of the trial outcomes.

### Sensitivity analysis

The results from the sensitivity analysis of all outcomes on a complete-case basis (that is, without imputation or adjustment for baseline predictors of missingness) were consistent with the results from the main analysis (Supplementary Tables S2 and S3). The results from the sensitivity analysis excluding participants with an HbA1c level of <8% (50.4% of the total sample) were similar to that of the main analysis, although statistical significance was not reached for HRQL and self-reported medication adherence outcomes (Supplementary Tables S4 and S5).

### Satisfaction with the intervention

Participants in the intervention group reported finding DiabeText valuable for diabetes management, with a perceived utility score of 8.1/10 (SD 2.47). Accessing messages was reported as being easy, with a mean accessibility score of 8.8/10 (SD 2.09). Overall satisfaction with DiabeText was high, scoring 8.1/10 (SD 2.37). Two participants felt the messages were intrusive and caused anxiety. No harm or adverse effects were reported by the remaining 332 participants in the intervention group who completed the final telephone interview (data not shown).

## Discussion

### Summary

This trial found that, over a 12-month period, DiabeText did not lead to statistically significant changes in glycaemic control, MPR, or lifestyle behaviour; nevertheless, it was associated with improved self-reported adherence to antidiabetic medication, diabetes self-efficacy, and HRQL.

### Strengths and limitations

This RCT contains a representative sample of people with T2DM for Spain, which supports the findings’ external validity in the Spanish health system. Notably, the average age of participants — namely, 65 years — closely mirrors that of the broader population with T2DM in Spain,^37^ thereby facilitating the application of results, despite the common challenge posed by older age in adopting digital interventions. Internal validity has been ensured through randomisation and the blinding of data collectors and the analyst. Although blinding participants was not feasible due to the nature of the intervention, this limitation may have a small impact on objectively measured outcomes, such as HbA1c level and MPR, because participants cannot directly change them.^38^ To assess medication adherence more effectively, both self-reported and electronic measurements were included; this dual approach helps to account for the inherent biases associated with each type of measurement.^39^,^40^

The primary limitation of this study is the challenge of accurately measuring participant adherence to the intervention because of uncertainties surrounding message comprehension and assimilation. Moreover, the intervention’s reach may have been further diluted when messages were delivered to participants’ caregivers, necessitating specific evaluation of this subgroup in future studies. It was also a limitation that there were no intermediate evaluations during follow-up, which could have aided the interpretation of results. Additionally, the low-intensity nature of the intervention could have influenced the absence of noticeable differences in diet and exercise between groups. It is advisable, therefore, to consider employing more sensitive instruments to gauge lifestyle changes.

### Comparison with existing literature

A recent meta-analysis of nine trials comparing SMS interventions with usual care for glycaemic control revealed not only a pooled effect on HbA1c of −0.37% (95% CI = −0.57 to −0.17; *P*<0.001), but also a clinically meaningful HbA1c reduction (>0.5%) in both the intervention and the control groups.^41^ The latter closely mirrors the findings presented here — namely, a −0.6% post-intervention reduction in both groups, suggesting a strong Hawthorne effect. Although some studies, such as that by Haider *et al*, reported similar results (–0.38% [95% CI = −0.53 to −0.23; *P*<0.001]),^42^ others like that by Zhang *et al* did not find statistically significant improvements (–0.09% [–0.43 to 0.25; *P* = 0.59]).^43^ These findings suggest that, although certain SMS interventions can effectively reduce HbA1c levels, not all yield such benefits. Some evidence suggests that interventions involving telemonitoring,^44^ medication adjustments,^45^ and primary care settings^46^ obtained superior reductions in HbA1c level. It has been reported, however, that multifaceted interventions are more likely to achieve better results in diabetes care.^47^

Available evidence shows that telehealth interventions are an effective method to increase medication adherence in patients with T2DM (0.501 [95% CI = 0.231 to 0.771; *P*<0.001])^48^ — including SMS interventions, which showed a pooled effect size of 0.36 (*P* = 0.001);^12^ this is similar to the results presented here on self-reported medication adherence. However, when adherence was estimated in terms of MPR, the authors of this study did not observe statistically significant differences between groups, as had previous authors.^49^,^50^ It is possible that including people with good adherence (MPR >82%) leaves little room for improvement, but it also reinforces the idea that dispensing medication from the pharmacy does not necessarily lead to such medication being taken. Regarding the observed sex differences in medication adherence, although some evidence suggests that women are more prone to non-adherence to antidiabetic medication,^51^ further research is needed to expand the evidence on gender differences in medication adherence improvements in response to digital interventions. The subgroup analysis indicated that DiabeText was associated with higher self-reported medication adherence in men but not in women. Previous studies suggest that women are more likely than men to experience challenges with adherence to antidiabetic medications.^51^ Future research with a stronger focus on gender perspectives is needed to explore how interventions such as DiabeText can more effectively support women in improving medication adherence.

DiabeText was associated with improvements in diabetes self-efficacy and HRQL. These outcomes are measured less frequently than physiological ones, with different instruments, and usually as secondary outcomes. Consequently, current evidence is still scarce and inconsistent, with some studies observing statistically significant improvements^52^,^53^ while others have not.^45^,^54^,^55^ However, a recent meta-analysis including four studies based on SMS interventions showed statistical improvements in self-efficacy with a large effect size of 1.46 (*P* = 0.006).^56^

Likewise, dietary and physical activity estimates are scarcely reported, and are measured with incomparable instruments. Although the authors of the study presented here observed a trend to improvement in their previous feasibility trial,^22^ it was not the case in the current effectiveness trial, which is consistent with findings from previous studies.^56^–^58^

### Implications for research and practice

In the UK and Spain, as well as in other countries, SMSs are increasingly being used by health service providers to send information about appointments or follow-ups to patients, as well as promoting participation in screening programmes.^59^ In the UK, over the last few years, GP surgeries have embraced text messaging as a key source of communication with patients.^60^ This growing interest in text messaging-based communication, plus the new technological infrastructures now being deployed for using SMSs, enormously facilitate the implementation of interventions such as DiabeText in routine health-service provision. As part of a new grant, the authors identified the key steps required for implementing DiabeText in primary healthcare centres; these are outlined in Box 1.

Box 1 **How could DiabeText be implemented in general practice? Proposed roadmap for implementing DiabeText as part of routine health service provision in primary healthcare centres**

- Packaging DiabeText in self-contained software (for example, a digital library containing the set of text messages plus the algorithms defining how, when, and which of these messages will be sent). This packaging allows having a technological product that can be protected (intellectual property) and transferred.
- Transferring DiabeText software to the HSO. This can be done either directly (from the research institution to the HSO) or indirectly (via a technological company that could then transfer it to the HSO). Regulatory requirements need to be met.
- Integrating DiabeText in the existing HSO technological information system. This may^a^ involve:
  — integration with the HSO’s EHR database (to allow personalisation of text messages based on routinely recorded clinical data);
  — integration with the HSO’s PROM tool (to allow personalisation of text messages based on patient-reported data); and
  — integration with the HSO’s SMS platform (to deliver SMSs to patients).

Ensuring patient information security is a key requisite. This could be achieved by installing the DiabeText software within the servers of the HSO, thereby avoiding patient information flowing outside of these servers.

- Implementing a system to allow primary healthcare professionals to prescribe DiabeText:
  — developing a system (application programming interface) by which DiabeText can be prescribed (activated) to eligible patients by primary healthcare providers during routine clinical practice; and
  — creating a culture of digital prescription of DiabeText among primary healthcare providers (for example, through training and reminders).
- Regularly updating the DiabeText software. This is key to ensure the information in the SMSs patients receive is aligned with latest guidelines for diabetes self-management.

aIntegration with EHR and with PROM systems may be particularly challenging (and unfeasible in some HSOs).

In terms of implications for research, as pointed out by a recent Cochrane systematic review,^47^ multicomponent interventions constitute the best approach to achieve meaningful population-level improvements across the majority of outcomes in people with T2DM. New studies are needed to determine the extent to which the observed benefits of mHealth interventions, such as DiabeText, could be maximised when combined with other evidence-based co-interventions, such as patient education, clinician education, or case management.

These findings highlight the potential of tailored mobile health interventions such as DiabeText to enhance medication adherence, self-efficacy, and quality of life among patients with T2DM. These improvements suggest that integrating similar interventions into routine primary care could provide valuable support for patient self-management, particularly for behavioural and psychosocial outcomes. However, the lack of significant impact on physiological measures such as HbA1c underscores the need for further research to refine and enhance these interventions to achieve broader clinical benefits.

## Supplementary Information



## Data Availability

The data that support the findings of this study are available from the corresponding author upon reasonable request.
