# Supplementary material for: Impact of an SMS intervention to support type 2 diabetes self-management: DiabeText clinical trial
Source: Br J Gen Pract. 2025 Jun 27;75(756):e457–65. doi: 10.3399/BJGP.2024.0206 (PMC11755587; doi:10.3399/BJGP.2024.0206)
Supplement: Supplementary file 1 [file BJGP.2024.0206_suppl.pdf]

Supplementary Table S1. Detailed description of the DiabeText intervention (TiDieR checklist).

<table>
<tr><th>Item</th><th></th><th>Description</th></tr>
<tr><td>1.</td><td>BRIEF NAME
Provide the name or a phrase that describes the intervention.</td><td>DiabeText is a mobile health Intervention to Support Diabetes Medication taking in adults with Type 2 Diabetes receiving antidiabetic treatment</td></tr>
<tr><td>2.</td><td>WHY
Describe any rationale, theory, or goal of the elements essential to the intervention.</td><td>To promote diabetes self-management including medication taking and healthy lifestyles in people with type 2 diabetes mellitus</td></tr>
<tr><td>3.</td><td>WHAT
Materials: Describe any physical or informational materials used in the intervention, including those provided to participants or used in intervention delivery or in training of intervention providers. Provide information on where the materials can be accessed (e.g. online appendix, URL).</td><td>Tailored short text messages (SMSs) sent to the mobile phones of adults with type 2 diabetes. Participants are grouped in three different profiles based on their lifestyle characteristics coming from the validated 14 items short screener for assessing Mediterranean Diet Adherence among adults (MEDAS-14)[1] and the International Physical Activity Questionnaire (IPAQ)[2].
<table>
<tr><th></th><th>MEDAS-14</th><th></th><th></th></tr>
<tr><td>IPAQ</td><td>Low adherence</td><td>Moderate adherence</td><td>High adherence</td></tr>
<tr><td>Low</td><td>Balance</td><td>Exercise</td><td>Exercise</td></tr>
<tr><td>Moderate</td><td>Diet</td><td>Balance</td><td>Exercise</td></tr>
<tr><td>High</td><td>Diet</td><td>Diet</td><td>Balance</td></tr>
</table>
Each of the three possible profiles receive a different number of SMSs about diet and exercise attending this proportion:
<table>
<tr><th></th><th>Medication</th><th>Diet</th><th>Physical Activity</th><th>Diabetes self-management</th></tr>
<tr><td>Balance</td><td>50%</td><td>20%</td><td>20%</td><td>10%</td></tr>
<tr><td>Diet</td><td>50%</td><td>30%</td><td>10%</td><td>10%</td></tr>
<tr><td>Exercise</td><td>50%</td><td>10%</td><td>30%</td><td>10%</td></tr>
</table></td></tr>
</table>

<table>
<tr><td></td><td></td><td>Some messages include hyperlinks to different online resources of interest to people with type 2 diabetes.
Both, text messages and linked online resources are in Spanish.
An informative sample of DiabeText text messages are available upon request to the corresponding authors.</td></tr>
<tr><td>4.</td><td>Procedures: Describe each of the procedures, activities, and/or processes used in the intervention, including any enabling or support activities.</td><td>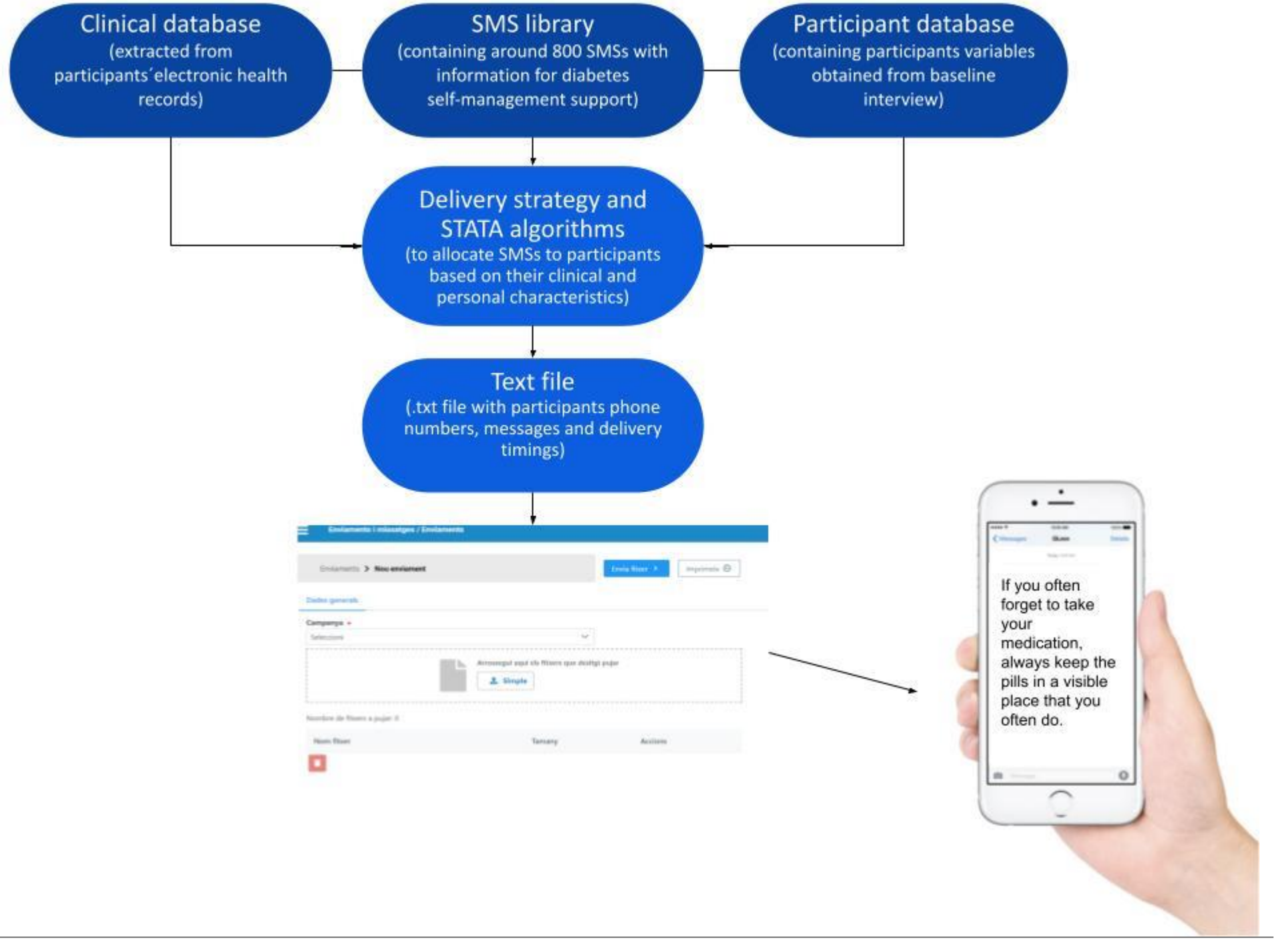
</td></tr>
</table>

		The DiabeText system is based on a set of algorithms that merge three input sources (clinical databases, database of brief messages, and patient-reported data) to generate text files (outputs) containing the text messages that each patient would receive on a certain day based on their clinical and behavioural data. The resulting .txt files are then manually uploaded to the SMS platform of the Balearic Islands Health Service which sends them to the patients.
5.	WHO PROVIDED<br>For each category of intervention provider (e.g. psychologist, nursing assistant), describe their expertise, background and any specific training given.	The DiabeText intervention is designed to work autonomously after physicians' prescription. In the research context, the enrolment of patients is assisted by the project staff.
6.	HOW<br>Describe the modes of delivery (e.g. face-to-face or by some other mechanism, such as internet or telephone) of the intervention and whether it was provided individually or in a group.	Individual short text messages (160 characters maximum) sent to mobile phones of participants
7.	WHERE<br>Describe the type(s) of location(s) where the intervention occurred, including any necessary infrastructure or relevant features.	DiabeText is a mobile health intervention without a specific location to be held. However, participants should remain in Spain during the study because the technological system is not able to send SMSs out of national borders without extra costs. The communication channel is unidirectional and asynchronous.
8.	WHEN and HOW MUCH<br>Describe the number of times the intervention was delivered and over what period of time including the number of sessions, their schedule, and their duration, intensity or dose.	Each participant receives one daily SMS between Monday to Friday during the first four months. Then, 3 SMS per week the following 4 months and after that, the frequency diminishes to 2 SMS per week for the last 4 months of the intervention period. Participant also receive extra SMS when new information is registered in their electronic health records about upcoming appointments in primary care, next drug dispensing at the pharmacy, blood test results for A1C and body weight.

9.	TAILORING<br>If the intervention was planned to be personalised, titrated or adapted, then describe what, why, when, and how.	The intervention is personalised based on data extracted from electronic health records (clinical data) and baseline interviews (behavioural data). First, based on patient-reported data during the baseline interview, participants are allocated to one of the three different profiles available (balance, diet, exercise), receiving a different number of SMSs about diet and exercise respectively -see point three above). Secondly, participants receive personalized SMSs according to the antidiabetic medication registered in their electronic health record. Third, the receive personalized SMSs depending on a list of variables of personalization (IMC, use of the internet on the mobile phone, diabetic foot, cholesterol higher than 200mg/dl, IPAQ score, smoking status, hypertension, chronic kidney disease, working status, Ramadan follow-up). Finally, the SMSs that had less than 130 characters include the name of the participant at the beginning.
10.	MODIFICATIONS<br>If the intervention was modified during the course of the study, describe the changes (what, why, when, and how).	No modifications were introduced in the DiabeText intervention during the study
11.	HOW WELL<br>Planned: If intervention adherence or fidelity was assessed, describe how and by whom, and if any strategies were used to maintain or improve fidelity, describe them.	Research staff checked that all participants are receiving the intervention correctly by using the *Bitmessage* platform (which keeps a record of the SMS successfully delivered). We also programmed the intervention to send messages to two phone numbers from the research team with anonymous data from two participants in the control group, to check that SMSs are correctly sent and received. At the end of follow-up, we asked all participants in the intervention group if they received the SMSs properly.
12.	Actual: If intervention adherence or fidelity was assessed, describe the extent to which the intervention was delivered as planned.	According to the meta-data extracted from the Bitmessage message platform, 66,993 (98.4%) out of the 68,092 SMS uploaded to the platform immediately reached participants' phones. 177 (0.26%) were put on hold (mainly due to poor mobile coverage) and delivered later, and 922 (1.35%) were not delivered (mainly because the operator could not send them or due to wrong number). Therefore, 318 out of 334 (95.2%) participants in the intervention group who completed the study received more than 90% of the intervention (>165 messages in one year). One participant received less than half of the intervention (received 62 messages in one year) and one who did not receive any message.
13.	Other important information about the intervention	- Theoretical framework: Behavioral change wheel<br>- Design process was based in the Medical Research Council guidance for development and evaluation of complex interventions and has been fully described in previously published work:

1. **Zamanillo-Campos R**, Fiol-DeRoque MA, Serrano-Ripoll MJ, Mira-Martínez S, Ricci Cabello I. Development and Evaluation of Diabetext, a Personalized Mhealth Intervention to Support Medication Adherence and Lifestyle Change Behaviour in Patients with Type 2 Diabetes in Spain: A Phase II Pragmatic Randomized Controlled Clinical Trial. *International Journal of Medical Informatics*. 2023.
2. **Zamanillo-Campos R**, Zaforteza-Dezcallar M, Boronat-Moreiro MA, Leiva-Rus A, Ripoll-Amengual J, Konieczna J, Fiol-DeRoque MA, Ricci Cabello I. Non-adherence to non-insulin glucose-lowering drugs: prevalence, predictors and impact on glycemic control and insulin initiation. A longitudinal cohort study in a large primary care database in Spain. *European Journal of General Practice*. 2023.
3. **Zamanillo-Campos R**, Serrano-Ripoll MJ, Taltavull-Aparicio JM, et al. Perspectives and Views of Primary Care Professionals Regarding DiabeText, a New mHealth Intervention to Support Adherence to Antidiabetic Medication in Spain: A Qualitative Study. *Int J Environ Res Public Health*. 2022;19(7):4237.
4. **Zamanillo-Campos R**, Serrano-Ripoll MJ, Taltavull-Aparicio JM, et al. Patients' Views on the Design of DiabeText, a New mHealth Intervention to Improve Adherence to Oral Antidiabetes Medication in Spain: A Qualitative Study. *Int J Environ Res Public Health*. 2022;19(3):1902.

Developers and owners: The researchers Ignacio Ricci-Cabello, Rocío Zamanillo-Campos, Maria Jesús Serrano-Ripoll, Elena Gervilla-García and Maria Antonia Fiol-deRoque from the Health Research Institute of the Balearic Islands.

EHRs; electronic health records, IPAQ; six-item International Physical Activity Questionnaire, MEDAS-14; 14-point Mediterranean diet adherence screener, SMSs; short text messages, A1c; glycated hemoglobin.

1. Schröder H, Fitó M, Estruch R, et al. A Short Screener Is Valid for Assessing Mediterranean Diet Adherence among Older Spanish Men and Women. J Nutr. 2011;141(6):1140-1145. doi:10.3945/jn.110.135566
2. Román Viñas B, Ribas Barba L, Ngo J, Serra-Majem L. Validación en población catalana del cuestionario internacional de actividad física. Gac Sanit. 2013;27(3):254-257. doi:10.1016/j.gaceta.2012.05.013

Supplementary Box S1. Details on outcomes' collection and calculations.

A. DATA EXTRACTED FROM ELECTRONIC HEALTH RECORDS

Glycated hemoglobin (HbA1c)

HbA1c was extracted as percentage which is calculated following the standard formulae [HbA1c(%)=(HbA1c(mmol/mol)+23.5)/ 10.93] based on its concentration in blood samples. It identifies average plasma glucose concentration (1).

The most recent HbA1c data registered between 6th of April and 12th of November in 2021 were extracted for all participants at baseline.

At post-intervention, we extracted the most recent data which was available between 21st September 2022 and 21st February 2023.

Medication possession ratio (MPR)

We calculated adherence in terms of medication possession ratio (MPR), defined as the number of days with treatment as medication being dispensed from the pharmacy to the patient (numerator), out of the total days of treatment prescribed by the doctor (denominator) (2):

MPR = [Days with treatment (prescription dispensed) / Days with treatment as prescribed by the doctor] x 100

At baseline it was calculated as the mean adherence for all the glucose lowering drugs prescribed during the 6 months previous to recruitment excluding insulin.

At post-intervention it was calculated as the mean adherence for all the glucose lowering drugs prescribed during the 12 months follow-up excluding insulin.

Medication adherence based on MPR

Adherence was stated as MPR≥80% and non-adherence was considered when MPR<80% (3,4).

B. DATA COLLECTED FROM TELEPHONE INTERVIEWS

Self-reported adherence to oral glucose lowering drugs

Self-reported adherence to glucose medications was measured with a 7-items ad hoc questionnaire adapted from Chaves-Torres et al. (5) for people with T2D. Participants who obtained 7 points were considered adherent while the ones with < 7 points were non-adherent.

Health-related quality of life

The 5-level EuroQol 5-dimensional questionnaire (EQ-5D-5L) questionnaire (6,7) was completed at baseline and post-intervention interviews. The index score was calculated using STATA syntax code and values with the Spanish value according to Ramos-Goñi JM et al. (8).

Self-efficacy to manage diabetes

The validated scale known as diabetes management self-efficacy scale in Spanish (DSES-S) was completed at baseline and post-intervention interviews. The score for each item was the number circled. If two consecutive numbers were circled, the lower number (less self-efficacy) was coded. If the numbers were not consecutive, the item was not scored. The score for the scale is the mean of the eight items. If more than two items were missing, we did not score the scale following instructions. Higher number indicates higher self-efficacy (9).

Adherence to Mediterranean Diet

The 14-point Mediterranean Diet Adherence Screener (MEDAS-14) (10) questionnaire was registered at baseline and post-intervention. Participants were classified as low adherents (≤5), moderate adherents (6 to 9 points) or high adherents (≥10 points).

Physical Activity

A 6-items adapted from the short version of the International Physical Activity Questionnaire (IPAQ) (11) was registered at baseline and post-intervention. Participants were classified as having a low, moderate or high level of physical activity based on metabolic equivalent of task (METs) calculation and the rules described below:

High

Any of the following 2 criteria:

• Vigorous physical activity on at least 3 days and accumulating at least 1500 MET-minutes/week, or

• 7 or more days of any combination of walking, moderate, or vigorous activities accumulating a minimum of 3000 MET-minutes/week.

Moderate

Any of the following 3 criteria:

• 3 or more days of vigorous activity of at least 20 minutes per day

• 5 or more days of moderate-intensity activity and/or walking at least 30 minutes per day, or

• 5 or more days of any combination of walking, moderate or vigorous activities achieving a minimum of at least 600 MET-minutes/week.

Low

• No reported activity

• Does not meet categories 2 or 3.

Participants' satisfaction with the intervention and potential related harms

We asked the following questions to participants in the intervention group at 12 months follow-up:

1. Based on your experience, do you think that receiving informational text messages about diabetes on your mobile phone is a useful tool to help you improve your diabetes care? Scale from 1 (Not at all useful) to 10 (Very useful)
2. Have you found it easy to access the messages you received? Scale from 1 (Not easy) to 10 (Very easy).
3. Have you enjoyed receiving information through your mobile phone during this time? Scale from 1 (Not enjoyed at all) to 10 (Totally enjoyed).
4. Do you think that receiving messages to improve diabetes management for 1 year has caused you any harm? Yes/No. If the answer is affirmative, describe the harm experienced.

1. What is HbA1c? – Definition, Units, Conversion, Testing & Control. https://www.diabetes.co.uk/what-is-hba1c.html. Accessed: 2024-02-09
2. Cramer JA. A Systematic Review of Adherence with Medications for Diabetes. Diabetes Care. 2004;27(5):1218-1224. doi: 10.2337/diacare.27.5.1218.
3. Krass I, Schieback P, Dhippayom T. Adherence to diabetes medication: a systematic review. Diabet Med. 2015 Jun;32(6):725-37. doi: 10.1111/dme.12651.
4. World Health Organization (WHO). Adherence to long-term therapies: evidence for action, 2003 – PAHO/WHO / Pan American Health Organization.

https://www.paho.org/en/documents/who-adherence-long-term-therapies-evidence-action-2003 Accessed: 2024-02-09

5. Chaves Torres NM, Echeverri Sarmiento JE, Ballesteros DA, Quijano Rodriguez J, Camacho D. Validación de la escala de Morisky de 8 ítems en pacientes con enfermedad renal crónica. *Revista Med*. 2016;24(2):23-32. doi:10.18359/rmed.2627
6. Herdman M, Gudex C, Lloyd A, et al. Development and preliminary testing of the new five-level version of EQ-5D (EQ-5D-5L). *Quality of Life Research*. 2011;20(10):1727-1736. doi:10.1007/s11136-011-9903-x
7. Cabasés JM. El EQ-5D como medida de resultados en salud. *Gac Sanit*. 2015;29(6):401-403. doi:10.1016/J.GACETA.2015.08.007
8. Ramos-Goñi JM, Craig B, Oppe M, Ramallo-Fariña Y, Pinto-Prades JL, Luo N, Rivero-Arias O. Handling data quality issues to estimate the Spanish EQ-5D-5L Value Set using a hybrid interval regression approach. Value in Health 2017. https://euroqol.org/eq-5d-instruments/eq-5d-5l-about/valuation-standard-value-sets/
9. Ritter PL, Lorig K, Laurent DD. Characteristics of the Spanish- and English-Language Self-Efficacy to Manage Diabetes Scales. *Diabetes Educator*. 2016;42(2):167-177. doi:10.1177/0145721716628648
10. Schröder H, Fitó M, Estruch R, et al. A Short Screener Is Valid for Assessing Mediterranean Diet Adherence among Older Spanish Men and Women. *J Nutr*. 2011;141(6):1140-1145. doi:10.3945/jn.110.135566
11. Román Viñas B, Ribas Barba L, Ngo J, et al. Validación en población catalana del cuestionario internacional de actividad física. *Gac Sanit*. 2013;27(3):254-257. doi:10.1016/j.gaceta.2012.05.013

Supplementary Table S2. Association between the DiabeText intervention and glycemic control, diabetes self-efficacy and quality of life (sensitivity analysis with not imputed data).

	Baseline		12 months follow-up		Association coefficient estimates for participants in the intervention group compared to controls		Effect size
	Control (n=371)	Intervention (n=371)	Control (n=340)	Intervention (n=334)	Beta (95%CI)	p-value[1]	Cohen's D (95%CI)
Glycemic control (Hba1c (%)), median (IR)	8 (7.6-8.8)	8.1 (7.7-8.7)	7.4 (6.7-8.3)[2]	7.5 (6.7-8.2)[2]	-0.026 (-0.078 to 0.130)	0.627	N/A
Diabetes self-efficacy scale (DSES), median (IR)	6.9 (5.9-7.9)	6.9 (5.8-8)	8 (6.6-8.9)	8.6 (7.4-9.3)	0.274 (0.112 to 0.436)	**0.001***	0.35 (0.20 to 0.50)
Quality of life (EQindex), median (IR)	0.93 (0.89-1)	0.93 (0.88-1)	0.97 (0.89-1)	1 (0.92-1)	0.018 (0.006 to 0.030)	**0.002***	0.18 (0.04 to 0.32)

Analyses were performed using linear mixed-effects models adjusted for baseline values.

HbA1c, glycated haemoglobin; IR, interquartile range; CI, 95% coefficient interval

[1]p values and 95%CI based on percentiles using non-parametric Bootstrap

[2] post-intervention HbA1c available for 321 patients in the control group and 309 in the intervention group

*Significance stated at p<0.05

Supplementary Table S3. Association between the DiabeText intervention and self-reported adherence to medication treatment, adherence to Mediterranean diet and adherence to physical activity (sensitivity analysis with not imputed data).

	Baseline		12 months follow-up		Association odds for participants in the intervention group compared to controls	
	Control (n=371)	Intervention (n=371)	Control (n=340)	Intervention (n=334)	OR (95% CI)[1]	p-value[2]
Self-reported adherence to antidiabetic medication, n (%)						
Non-adherent	177 (47.7%)	166 (44.7%)	143 (42.1%)	114 (34.1%)	1	
Adherent	194 (52.3%)	205 (55.3%)	197 (57.9%)	220 (65.9%)	1.372 (1.00 to 1.86)	**0.045***
Adherence to Mediterranean diet, n (%)						
Non-adherent	90 (24.3%)	100 (27.0%)	171 (50.3%)	163 (48.8%)	1	
Adherent	281 (75.7%)	271 (73.0%)	169 (49.7%)	171 (51.2%)	1.08 (0.79 to 1.47)	0.618
Adherence to physical activity, n (%)						
Non-adherent (low level)	126 (34.0%)	131 (35.3%)	146 (43%)	163 (48.8%)	1	
Adherent (moderate or hight level)	245 (66.0%)	240 (64.7%)	194 (57%)	171 (51.2%)	0.80 (0.58 to 1.12)	0.206

Analyses were performed using logistic regression adjusted for baseline values.

[1] data represents Odds Ratio (OR) with 95% confidence intervals in parentheses (CI)

[2] p values and 95%CI based on percentiles using non-parametric Bootstrap

*Significance stated at p<0.05

Supplementary Table S4. Association between the DiabeText intervention and glycemic control, adherence to medication treatment, diabetes self-efficacy and quality of life (sensitivity analysis excluding patients with HbA1c< 8%.).

	Baseline		12 months follow-up		Association coefficient estimates for participants in the intervention group compared to controls		Effect size
	Control (n=182)	Intervention (n=192)	Control (n=161)	Intervention (n=167)	Beta (95%CI)	p-value[1]	Cohen's D (95%CI)
Glycemic control (Hba1c (%)), median (IQR)	8.8 (8.3 to 9.7)	8.7 (8.3 to 9.4)	7.6 (6.9 to 8.6)[2]	7.6 (6.8 to 8.5)[2]	-0.097 (-0.367 to 0.172)	0.478	N/A
Adherence to medication treatment (MPR (%)), median (IQR)	95.1 (77.9 to 98.9)	94.8 (76.7 to 99.1)	89.4 (68.8 to 96.6)[3]	87.9 (74.3 to 96.3)[3]	-0.974 (-2.355 to 4.304)	0.566	N/A
Diabetes self-efficacy scale (DSES), median (IQR)	7.0 (5.9 to 7.9)	6.8 (5.5 to 7.9)	8.0 (6.5 to 8.8)	8.4 (6.8 to 9.3)	0.380 (0.048 to 0.711)	**0.025***	0.24 (0.03 to 0.45)
HRQL (EQ-5D index), median (IQR)	0.92 (0.89 to 1.00)	0.92 (0.87 to 1.00)	1.00 (0.92 to 1.00)	0.98 (0.89 to 1.00)	-0.004 (-0.021 to 0.014)	0.687	N/A

Analyses were performed using linear mixed-effects models adjusted for baseline values with MI BOOT estimations.

HbA1c, glycated haemoglobin; IQR, interquartile range; HRQL, health-related quality of life; CI, 95% coefficient interval; N/A, not applicable

[1] p values and 95%CI based on percentiles using non-parametric Bootstrap

[2] post-intervention HbA1c available for 159 patients in the control group and 152 in the intervention group

[3] post-intervention MPR available for 182 patients in the control group and 192 in the intervention group, so no imputations were necessary

* p<0.05

Supplementary Table S5. Association between the DiabeText intervention and medication possession ratio, self-reported adherence to antidiabetic medication, adherence to Mediterranean diet and adherence to physical activity (sensitivity analysis excluding patients with HbA1c< 8%.)

	Baseline		12 months follow-up		Association odds for participants in the intervention group compared to controls	
	Control (n=182)	Intervention (n=192)	Control (n=161)	Intervention (n=167)	OR (95% CI)[1]	p-value[2]
**Medication Possession Ratio[3], n (%)**						
Non-adherent (MPR < 80%)	49 (26.9%)	56 (29.2%)	63 (34.6%)	68 (35.4%)	1	
Adherent (MPR ≥ 80%)	133 (73.1%)	136 (70.8%)	119 (65.4%)	124 (64.6%)	1.005 (0.620 to 1.628)	0.984
**Self-reported adherence to antidiabetic medication, n (%)**						
Non-adherent	88 (48.4%)	89 (46.4%)	74 (46.0%)	63 (37.7%)	1	
Adherent	94 (51.7%)	103 (53.7%)	87 (54.0%)	104 (62.3%)	1.395 (0.911 to 2.138)	0.125
**Adherence to Mediterranean diet, n (%)**						
Non-adherent	53 (29.1%)	58 (30.2%)	82 (50.9%)	87 (52.1%)	1	
Adherent	129 (70.9%)	134 (69.8%)	79 (49.1%)	80 (47.9%)	0.993 (0.660 to 1.493)	0.973
**Adherence to physical activity, n (%)**						
Non-adherent (low level)	60 (32.4%)	68 (35.4%)	62 (38.5%)	83 (49.7%)	1	
Adherent (moderate or high level)	122 (67.0%)	124 (64.6%)	99 (61.5%)	84 (50.3%)	0.673 (0.432 to 1.049)	0.081

Analyses were performed using logistic regression adjusted for baseline values with MI BOOT estimations.

[1] data represents Odds Ratio (OR) with 95% confidence intervals in parentheses (CI)

[2] p values and 95%CI based on percentiles using non-parametric Bootstrap

[3] post-intervention MPR available for 182 patients in the control group and 192 in the intervention group, so no imputations were necessary

*Statistically significance stated at p<0.05
